# Heterogeneity of d-glucuronyl C5-epimerase expression and epigenetic regulation in prostate cancer

**DOI:** 10.1002/cam4.108

**Published:** 2013-08-05

**Authors:** Tatiana Y Prudnikova, Nikolaos Soulitzis, Olesya S Kutsenko, Lyudmila A Mostovich, Klas Haraldson, Ingemar Ernberg, Vladimir I Kashuba, Demetrios A Spandidos, Eugene R Zabarovsky, Elvira V Grigorieva

**Affiliations:** 1 Institute of Molecular Biology and Biophysics SD RAMSNovosibirsk, Russia; 2 Medical School of University of CreteHeraklion, Greece; 3 MTC, Karolinska InstituteStockholm, Sweden; 4 Institute of Molecular Biology and GeneticsKiev, Ukraine

**Keywords:** d-Glucuronyl C5-epimerase, expression, heparansulfate proteoglycan, heterogeneity, methylation, prostate cancer

## Abstract

Heparansulfate proteoglycans (HSPG) play an important role in cell–cell and cell–matrix interactions and signaling, and one of the key enzymes in heparansulfate biosynthesis is d-glucuronyl C5-epimerase (GLCE). A tumor suppressor function has been demonstrated for *GLCE* in breast and lung carcinogenesis; however, no data are available as to the expression and regulation of the gene in prostate cancer. In this study, decreased *GLCE* expression was observed in 10% of benign prostate hyperplasia (BPH) tissues and 53% of prostate tumors, and increased GLCE mRNA levels were detected in 49% of BPH tissues and 21% of tumors. Statistical analysis showed a positive correlation between increased GLCE expression and Gleason score, TNM staging, and prostate-specific antigen (PSA) level in the prostate tumors (Pearson correlation coefficients GLCE/Gleason = 0.56, *P* < 0.05; GLCE/TNM = 0.62, *P* < 0.05; and GLCE/PSA = 0.88, *P* < 0.01), suggesting GLCE as a candidate molecular marker for advanced prostate cancer. Immunohistochemical analysis revealed an intratumoral heterogeneity of GLCE protein levels both in BPH and prostate cancer cells, resulting in a mixed population of GLCE-expressing and nonexpressing epithelial cells in vivo. A model experiment on normal (PNT2) and prostate cancer (LNCaP, PC3, DU145) cell lines in vitro showed a 1.5- to 2.5-fold difference in *GLCE* expression levels between the cancer cell lines and an overall decrease in GLCE expression in cancer cells. Methyl-specific polymerase chain reaction (PCR), bisulfite sequencing, and deoxy-azacytidin (aza-dC) treatment identified differential GLCE promoter methylation (LNCaP 70–72%, PC3 32–35%, DU145, and PNT2 no methylation), which seems to contribute to heterogeneous GLCE expression in prostate tumors. The obtained results reveal the complex deregulation of *GLCE* expression in prostatic diseases compared with normal prostate tissue and suggest that GLCE may be used as a potential model to study the functional role of intratumor cell heterogeneity in prostate cancer progression.

The molecular mechanisms of intratumour heterogeneity of cancer cells, contributing to tissue malignisation, remain unclear. This study reveals the complex deregulation of d-glucuronyl C5-epimerase (GLCE) expression in benign prostatic hyperplasia and prostate tumours, and the high intratumour heterogeneity of prostate cancer cells in terms of GLCE expression and promoter methylation. The results suggest that GLCE may be used as a potential target gene to study the functional role of cancer cell heterogeneity in disease progression and treatment.

## Introduction

d-Glucuronyl C5-epimerase (GLCE) plays an important role in heparansulfate proteoglycan (HSPG) biosynthesis, catalyzing the epimerization of d-glucuronic acid (GlcA) to l-iduronic acid (IdoA) in polysaccharide HS chains 1,2. l-IdoA-containing motifs increase the flexibility of the HS molecules, required for their interaction with growth factors and other protein ligands 3. The presence of l-IdoA seems to be inherent for various organisms from mammals to prokaryotes, where multiple candidate C5-epimerases were revealed by an in silico screen, explaining the presence of l-IdoA in bacterial and archaeal cell wall polysaccharides 4. High *GLCE* conservatism over almost all known species (84–86% homology for nucleic acid and 95–98% homology for aminoacid sequences) and neonatal lethality of the Glce(−/−) mice upon targeted disruption of a murine glucuronyl C5-epimerase gene 5 further support the principal importance of the gene in normal cell physiology and different pathological processes.

It has been shown that Glce activity is an important determinant of dorsoventral axis formation and patterning in zebrafish 6 and lymphoid organ development in mice 7. Glce deficiency impairs B-cell maturation and differentiation, resulting in decreased plasma cell numbers and immunoglobulin levels 8. Single-nucleotide polymorphisms (SNPs) in GLCE are associated with triglyceride and high-density lipoprotein cholesterol (HDL-C) levels in Turks, and mouse studies support a role for Glce in lipid metabolism 9.

Recently, a direct involvement of GLCE in carcinogenesis was shown for breast and lung cancer. GLCE expression is significantly decreased in breast tumors 10 and lung cancer cell lines 11, and its restoration suppresses cancer cell proliferation in vitro and tumor growth in vivo, suggesting a tumor suppressor function for GLCE in breast and small-cell lung cancers 11,12. However, in spite of the similar antiproliferative action of GLCE in the examined tissues, its molecular mechanisms in breast and lung cancers differ. Whereas the antiproliferative effect of GLCE on breast cancer cells may be associated with the enhanced expression of tumor suppressor genes and apoptosis-related genes 11, in lung cancer, these effects are mediated via the downregulation of several pro-angiogenic growth factors and their receptors 12. These results indicate that the functional role and the molecular mechanisms behind the GLCE effects may be tissue specific, and additional studies on other cancer types would be relevant.

In this study, GLCE expression in prostate tumors and prostate cancer cell lines and its epigenetic regulation by GLCE promoter hypermethylation were examined.

## Material and Methods

### Patients and tissue samples

All tissue samples were obtained from benign hyperplasia tissues (BPH) or primary prostate tumors during radical surgery at the Medical School of University of Crete (Heraklion, Greece) and Central Municipal Hospital N1 (Novosibirsk, Russia). The majority of patients were at the II-III stage of malignancy progression according the TNM staging system. PSA was 0.1–8.5 ng/mL for BPH and 1.8–50 ng/mL for adenocarcinoma patients, Gleason scores 2–9. Normal prostate tissue samples were obtained from normal prostates surgically resected by medical indications during nonprostate surgery. All patients provided written informed consent and the study protocol was approved by the Local Ethics Committees in accordance with the Helsinki Declaration of 1975.

### Cell lines, cell culture, and 5-aza-dC/TSA treatment

The human prostate cancer cell lines, LNCaP, PC3, and DU145, were obtained from MTC (Karolinska Institute, Stockholm, Sweden). The PNT2 normal human prostate epithelial cell line was obtained from the European Collection of Cell Cultures (ECACC, Salisbury, U.K.). All cell lines were maintained in RPMI medium supplemented with 2 mmol/L l-glutamine, 100 units penicillin, 100 μg/mL streptomycin, and 10% (v/v) fetal bovine serum at 37°C in a humidified 5% CO_2_ incubator. Treatment with deoxyazacytidine (5-aza-dC, 1 or 2 μg/mL) was performed by incubating the cells with the drugs for 72 h.

### Analysis of *GLCE* expression using multiplex RT-PCR

Multiplex real time (RT-PCR) analysis of *GLCE* expression was performed as previously described 11. Total RNA was extracted using the PureLink Total RNA Purification System (Invitrogen, Carlsbad, CA), cDNA was synthesized using a First Strand cDNA Synthesis kit (Fermentas, Hanover, MD). The PCR primers used were GLCE-F, 5′-AAGGGAGACGAGAGGGGAACGAA-3′; GLCE-R, 5′-GCCACCTTTCTCATCCTGGTTC-3′; GAPDH-F, 5′-GGGCGCCTGGTCACAA-3′; GAPDH-R, 5′-AACATGGGGGCATCAGCAGA-3′.

### Analysis of *GLCE* expression by quantitative TaqMan-based real-time RT-PCR

Quantitative RT–PCR (qRT–PCR) was performed as described 11 using the BioRad IQ5 Multicolor Real-Time PCR Detection System (BioRad, Hercules, CA) and the *GLCE* TaqMan Custom Assay (Applied Biosystems, Foster City, CA). The PCR primers and probes used were GLCE-F, 5′-TTCCAAAGTCTATGCACAGAGAGC-3′; GLCE-R, 5′-TCCACATTGTAGCCTTCAAAAGACA-3′; GLCE probe, 5′-FAM-CCCCTATCACCCCGATGGT-TAMRA-3′; *β*-actin-F, 5′-GGCACCCAGCACAATGAAG-3′; *β*-actin-R, 5′-GCCGATCCACACGGAGTACT-3′; *β*-actin-probe, 5′-FAM-TCAAGATCATTGCTCCTCCTGAGCGC-TAMRA-3′.

### Genomic DNA isolation and bisulfite conversion

Genomic DNA was isolated from the tissue samples using the E.Z.N.A. DNA isolation kit and bisulfite conversion of the genomic DNA was performed using an E.Z.N.A. DNA methylation kit (Zymo Research, Irvine, CA) according to the manufacturer's instructions.

### Methyl-specific PCR

Methyl-specific PCR for GLCE fragment amplification was carried out with primers specific for the methylated (M) and unmethylated (U) DNA sequences within *GLCE* CpG islands. The PCR primers were M-F, 5-TTGGTCGTAGTAGATTTCGAGTTTTGTC-3′; M-R, 5-CGCGCAACCGAAAAACCG-3′; U-F, 5-TTGAGTTTTGTTGTTTGTTTTGTAGTT-3′; U-R, 5-TATAAAAAAAACCCTCCCACTCCA-3′.

### Bisulfite sequencing

Amplification of the *GLCE* DNA fragment for bisulfite sequencing was performed using BS1 and BS2 primers (GLCE-BS1-F, 5′-GTATTTTAATAATGGTGTTTTGTTTGAG-3′; GLCE-BS1-R, 5′-CCAAAAATAATAAAAAACAATAAACTTTC-3′; GLCE-BS2-F, 5′-GAAAGTTTATTGTTTTTTATTATTTTTGGT-3′; GLCE-BS2-R, 5′-ACCCCCAAAATCCCTAATACATTAC-3′). The PCR products were purified using a DNA Clean and Concentrator Kit (Zymo Research), cloned into a TOPO-vector using a TOPO TA Cloning Kit for Sequencing (Invitrogen), and plasmid DNA was isolated using a Zyppy Plasmid Miniprep Kit (Zymo Research). Eight to ten clones were analyzed for each sample.

### Immunostaining

For immunohistochemistry, 5- to 6-μm sections of formalin-fixed, paraffin-embedded tissue sections were deparaffinized and antigen was retrieved by treatment with unmasking solution at 95–98°C for 20 min. For immunocytochemistry, cells were grown on glass coverslips and then fixed with phosphate-buffered 4% formaldehyde. The anti-GLCE primary antibody (1:300) was used for immunostaining (1 h at 37°C) and staining patterns were visualized with TexasRed-conjugated antibody against rabbit IgGs (1:1000, 30^ ^min at 37°C).

### Statistical analysis

Statistical analyses were performed using a computer program OriginPro 8.1; a value of *P* < 0.05 was considered to indicate a statistically significant difference. Data are expressed as the means ± SEM. Pearson correlation coefficients (*r*) were calculated to determine the association between GLCE expression and clinical parameters (PSA, Gleason, TNM).

## Results

### *d-Glucuronyl C5-epimerase* expression in BPH tissues and prostate tumors

**Figure 1:**
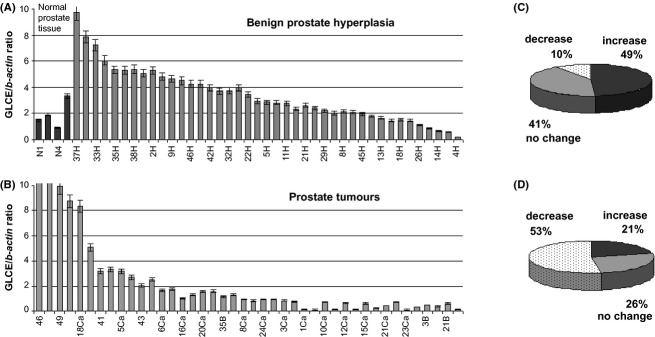
GLCE expression in normal human prostate tissue, benign prostate hyperplasia, and prostate tumors. (A, B) Real-time RT-PCR analysis of GLCE expression. Intensity of the amplified *GLCE* DNA fragments normalized to that of *b-actin*. Bars represent the mean ± SD from triplicate experiments (OriginPro 8.1). (C, D) Summary diagrams for the clinical sample distribution according the GLCE mRNA levels changes compared with the average GLCE level in normal prostate tissues (1.92 ± 1.04). A 50% cutoff value was considered significant.

d-Glucuronyl C5-epimerase expression was determined in normal human prostate tissue, BPH, and prostate tumors using TaqMan-based Real-Time and multiplex RT-PCR analyses (Fig. 1).

According to the obtained results, the GLCE mRNA expression levels in the majority of the BPH samples (90%) were at levels similar or even higher than those in the normal prostate tissue samples (average GLCE/b-actin ratio 1.92 ± 1.04) (Fig. 1). However, a significant decrease in GLCE expression was observed in 53% of the prostate tumors compared with the GLCE expression level in normal prostate tissue, and another 47% expressed GLCE at the normal or elevated levels. Similar results were obtained for two different tissue specimen sets, collected from Greek or Russian populations and independently investigated by TaqMan-based quantitative Real-Time or multiplex RT-PCR analyses, respectively.

Statistical analysis of an association between GLCE expression level and clinical data revealed no correlation between GLCE expression and age in any patients groups (BPH, early or advanced prostate cancer). However, in the BPH patient group with increased GLCE expression, tendency to a moderate positive correlation between GLCE expression and PSA level was observed (Pearson correlation coefficient *r* = 0.46, *P* < 0.07). In the prostate cancer samples, increased GLCE expression was positively associated with advanced disease (Pearson correlation coefficients GLCE/Gleason = 0.56, *P* < 0.05; GLCE/TNM = 0.62, *P* < 0.05; and GLCE/PSA = 0.88, *P* < 0.01), while no evident correlation was shown between GLCE expression and the clinical parameters for the prostate tumors both with normal and decreased GLCE expression (Table 1). The obtained results clearly showed a high positive correlation between GLCE expression and PSA level, Gleason score and TNM classification in the prostate tumors with increased GLCE expression, suggesting GLCE as a candidate molecular marker for advanced prostate cancer and potential target for new therapies.

**Table:** Statistical analysis of an association of GLCE expression with the patients' clinical data in BPH and prostate cancer

BPH		Prostate cancer			
GLCE decreased (0–0.96)		GLCE decreased (0–0.96)			
GLCE/b-actin ratio	0.56 ± 0.27	GLCE/b-actin ratio	0.50 ± 0.31
PSA, ng/mL	2.19 ± 1.44	PSA, ng/mL	10.67 ± 9.79
GLCE/age *r* = 0.09 GLCE/PSA *r* = −0.85		GLCE/age *r* = −0.18 GLCE/Gleason *r* = 0.14 GLCE/TNM *r* = 0.01 GLCE/PSA *r* = 0.02			
GLCE normal (0.96–2.88)		GLCE normal (0.96–2.88)			
GLCE/b-actin ratio	2.10 ± 0.53	GLCE/b-actin ratio	1.69 ± 0.54
PSA, ng/mL	2.86 ± 2.28	PSA, ng/mL	13.59 ± 10.61
GLCE/age *r* = 0.15 GLCE/PSA *r* = 0.38		GLCE/age *r* = −0.14 GLCE/Gleason *r* = −0.23 GLCE/TNM *r* = 0.10 GLCE/PSA *r* = −0.32			
GLCE increased (>2.88)		GLCE increased (>2.88)			
GLCE/b-actin ratio	4.90 ± 1.76	GLCE/b-actin ratio	6.78 ± 3.56
PSA, ng/mL	1.79 ± 1.10	PSA, ng/mL	12.25 ± 8.55
GLCE/age *r* = 0.04 GLCE/PSA *r* = 0.46		GLCE/age *r* = 0.09 GLCE/Gleason *r* = 0.56 GLCE/TNM *r* = 0.62 GLCE/PSA *r* = 0.88			

GLCE expression data and PSA levels are expressed as the mean ± SD, patient groups are formed according the GLCE mRNA levels changes compared with the average GLCE level in normal prostate tissues (GLCE/b-actin ratio 1.92 ± 1.04), a 50% cutoff value was considered significant. *r* – Pearson correlation coefficient, *P* < 0.05 was considered as a statistically significant difference (OriginPro 8.1). BPH, benign prostate hyperplasia; PSA, prostate-specific antigen.

**Figure 2:**
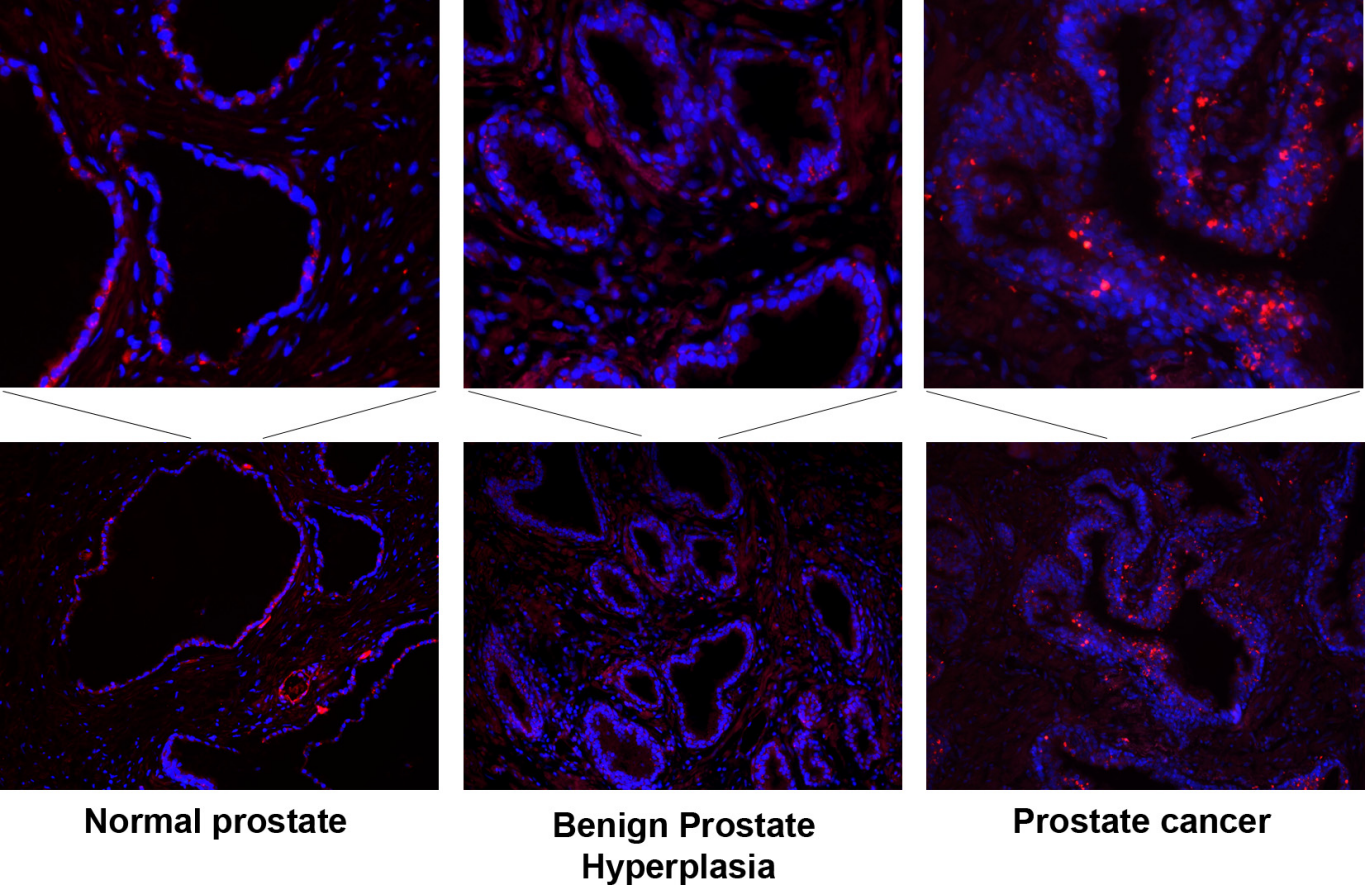
Immunohistochemical staining patterns for GLCE in normal human prostate, benign prostate hyperplasia, and prostate cancer tissues. Upper panel shows enlarged images of the areas with heterogeneous GLCE expression. Immunocytochemical staining with anti-GLCE primary antibody visualized with a TexasRed-conjugated secondary antibody (red color).

Immunohistochemical staining for GLCE protein content revealed also changes in the localization pattern for GLCE expression in pathological prostate tissues (Fig. 2).

GLCE was ubiquitously expressed in normal prostate epithelial cells, while BPH or prostate cancer epithelial cells showed a high heterogeneity in terms of GLCE expression levels. In BPH tissues, GLCE expression was completely lost or significantly increased in various epithelial cells; however, staining was still associated with the basal epithelial cell layer and delineated prostate tissue morphology. In prostate cancer tissues, GLCE-expressing cells were not associated with the basal epithelial layer and displayed even higher heterogeneity of the GLCE expression levels. Possibly, some genetic or epigenetic mechanisms deregulate the GLCE expression in various prostate epithelial cells, resulting in the appearance of a heterogeneous population of cells with different phenotypes and different malignant potential.

To verify the hypothesis that the differences in GLCE expression levels resulted from (or result in) the individual morphology of the epithelial cells inside the tumor, model experiments in vitro on morphologically different prostate cell lines were conducted.

### *d-Glucuronyl C5-epimerase* expression and promoter methylation in human prostate cancer cell lines

**Figure 3:**
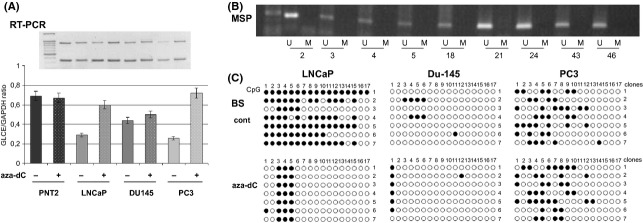
Methylation of *GLCE* promoter-associated CpG islands in human prostate tumors and cancer cell lines. (A) *GLCE* expression in different prostate cancer cell lines and its activation by treatment with 5-aza-deoxycytidine. The intensity of the amplified *GLCE* DNA fragments was normalized to that of *GAPDH*. Bars represent the mean ± SD from triplicate experiments (OriginPro 8.1). Upper panel – representative RT-PCR electrophoregram, aza-dC – 5-aza-deoxycytidine. (B) Methylation-specific PCR for the GLCE promoter region in prostate tumors. Two to 46 – prostate tumors, M and U – primers for methylated or unmethylated DNA sequences, respectively. (C) Bisulfite sequencing of prostate cancer cell lines LNCaP, DU145, and PC3. Seven different *E. coli* clones (1–7) were sequenced for each cell line, open and black circles – nonmethylated and methylated CpG dinucleotides, respectively.

GLCE expression was determined in normal human prostate epithelial cells (PNT2) and hormone-dependent (LNCaP) or hormone-independent (PC3, DU145) prostate adenocarcinoma cells using multiplex and real-time RT-PCR analyses. The examined prostate carcinoma cell lines showed *GLCE* mRNA levels 2- to 3-fold lower than the PNT2 normal prostate epithelial cells, whereas, 1.5- to 2.5-fold differences in GLCE expression were also observed between the cell lines (Fig. 3A).

The significant decrease in GLCE protein levels in prostate cancer cells was verified by immunocytochemical staining using a custom anti-GLCE polyclonal antibody (not shown).

Treatment of the cells with DNA-demethylating agent 5-aza-deoxycytidine (aza-dC) did not affect the *GLCE* mRNA level in the PNT2 and DU145 cells but increased the level in the LNCaP (2-fold) and PC3 cells (3-fold), suggesting the possible involvement of promoter methylation in the differential expression of GLCE in prostate cancer cells. According to the bisulfite sequencing results, all examined prostate cancer cell lines differed in terms of the functional contribution of GLCE promoter methylation in the regulation of the expression level of the gene: significant promoter methylation (70–72%) which was reduced by aza-dC was observed for the LNCaP cells and no methylation was observed for the DU145 cells. The most complex results were observed for the PC3 cell line, where aza-dC did not reduce the intermediate methylation levels (30–35%) in the GLCE promoter but increased GLCE expression, possibly through other regulatory mechanisms (Fig. 3B). Overall, all examined prostate cancer cell lines differed in terms of promoter methylation and its contribution to the regulation of GLCE expression. Possibly, a common imbalance of molecular mechanisms controlling GLCE expression results in the elevated heterogeneity of the cell population, with the subsequent clonal selection of the certain cancer cell subtype.

Of note, MSP analysis and bisulfite sequencing of the GLCE promoter region in prostate tumors revealed no GLCE promoter methylation in vivo (Fig. 3C). This suggests that either promoter methylation does not play a key role in GLCE regulation in vivo and that other mechanisms overpower its effect, or clonal selection is directed toward the elimination of methylation-regulated cancer cell subtypes in favor of methylation-independent variants of prostate cancer cells.

In conclusion, the obtained results show high intratumor heterogeneity of GLCE expression in prostate cancer cells in vivo and cell lines in vitro, which could be determined by the different extent of GLCE promoter methylation in the cancer cell population.

## Discussion

According to the obtained results, normal prostate, BPH, and prostate cancer differ in terms of GLCE expression, which was decreased in 10% of the BPH samples and 53% of the tumor samples. The results differ from those for benign and malignant breast tumors, where 36% and 82–84% of the tested samples revealed significantly decreased GLCE expression 10, suggesting a possible tissue specificity of GLCE regulation. Moreover, different molecular mechanisms seem to underlie the opposite changes in GLCE expression in the benign and malignant pathology of prostate tissue. In contrast to breast cancer (where almost no GLCE upregulation was observed), prostate pathological tissues were characterized by a predominant increase in GLCE expression in 49% of the BPH tissues and a decrease in GLCE expression in 53% of the prostate tumor tissues. Unexpectedly, the decreased GLCE expression was not associated with any main clinical signs of prostate cancer (Gleason score, TNM classification, PSA level), while exactly the increase in GLCE expression (21% of the prostate tumors) was associated with more aggressive disease. From the obtained results, it can be hypothesized that both GLCE expression deteriorations could be actively associated with a different prostate pathology.

The hypothesis is indirectly supported by the observation that maximal ectopic GLCE expression in morphologically different cell lines (breast carcinoma MCF7, small-cell lung cancer U2020, prostate cancer cells LNCaP, PC3) was not more than that in the corresponding normal cells 11,12. Possibly, other tight regulatory mechanism(s) govern GLCE expression and protect the cells from GLCE overproduction. Elevated GLCE levels in BPH (49% of the patients) and prostate cancer (21% of the patients) could indicate a disruption of the restricting mechanism(s) and a specific type of GLCE expression disorganization in this pathology, while a decrease in GLCE expression may be associated with specific prostate cancer subtypes.

A key point for the hypothesis is a basic principle of a gene regulation as a “mono-” or “multiregulated” gene. In “mono-regulated” gene mode, the only molecular mechanism (methylation, chromatin structure, or transcription factor[s] etc.) regulates the gene expression level, and its abrogation will uniformly alter the gene expression in the affected cells. This is an extremely oversimplified assumption and possibly “monoregulation” does not exist at all. In “multiregulated” gene mode, a reactivity of the different regulatory mechanisms to incoming signals will be multiplied by their combination and result in heterogeneous gene expression levels in cells. For example, both variable CpG site promoter methylation and histone modifications contribute to *TIMP3* downregulation in prostate cancer 13. *TIMP3* hypermethylation was only observed in DU145 cells but not LNCaP and PC3, where *TIMP3* expression could be upregulated by the combination of histone methylation inhibitor and TSA. These findings not only highlight the complex heterogeneity of epigenetic silencing in prostate cancer, but also suggest that tumor- and gene-specific alterations could be used to predict patients' response to epigenetic drugs 13. GLCE is another a multiregulated gene, where *GLCE* expression in breast cancer is controlled through complex molecular mechanisms, including at least chromatin structure, TCF4/b-catenin complex, and microRNA-218, but not GLCE promoter methylation 14–17. However, in prostate tissues, GLCE promoter methylation occurs in certain morphological subtypes of prostate cancer epithelial cells and contributes to the overall inactivation of the gene in the cells. Thus, at least four known “players” could contribute to the regulation of GLCE expression, and the balance between them will determine the GLCE expression level in every single prostate cancer cell and serve as a foundation for the high heterogeneity of the cells inside a tumor.

The obtained results stay in line with a growing body of experimental evidence that many types of tumors are organized in a hierarchy of heterogeneous cell populations 18. It has become exceedingly apparent that the intratumoral genetic and epigenetic heterogeneity is at the origin of tumor progression and it is also the byproduct of the selection process during progression, contributing to both cancer growth, and therapy tolerance 19,20. A new plastic cancer stem cell theory of cancer development was suggested, based on the high heterogeneity of cancer cells within a tumor and combining two established models of cancer development and progression (the clonal evolution and cancer stem cell models [CSC]) 21.

Prostatic adenocarcinoma is an epithelial malignancy characterized by marked histological heterogeneity although there are limited data available on the involvement of specific genes. Heterogeneous expression or genomic rearrangements have been shown for PTEN loss 22, telomerase activity 23, ras oncogene activation, and p53 tumor suppressor gene mutations 24. The highly heterogeneous nature of prostate cancer provides a real challenge for clinical disease management and a detailed understanding of the genetic and epigenetic alterations in all cells, including small subpopulations, would be highly advantageous 25.

The obtained results on the complex deregulation of *GLCE* expression and promoter methylation in prostate pathology suggest that GLCE may be a gene of interest in the study of the functional role of intratumor cell heterogeneity in prostate cancer progression and treatment.
